# Supplementary material for: Production of Reactive Oxygen Species by Epicardial Adipocytes Is Associated with an Increase in Postprandial Glycemia, Postprandial Insulin, and a Decrease in Serum Adiponectin in Patients with Severe Coronary Atherosclerosis
Source: Biomedicines. 2022 Aug 22;10(8):2054. doi: 10.3390/biomedicines10082054 (PMC9405686; doi:10.3390/biomedicines10082054)
Supplement: Supplementary file 1 [file biomedicines-10-02054-s001.zip › biomedicines-1860765-supplementary.pdf]

Table S7. Comparison of clinical and biochemical characteristics of patients with Gensini score more or less than 70.55 points.

Parameters	Group A Patients with Gensini score <70.55 (n=11)	Group B Patients with Gensini ≥ 70.55 (n=8)	P
Gender (m/f), n (%)	7(64) / 4(36)	7(87.5) / 1(12.5)	>0.05
Age, years, Me (max-min)	62 (53-72)	64 (53-71)	>0.05
Diabetes mellitus/Prediabetes, n (%)	4 (36.4)	7 (87.5)	**0.0258**
BMI, kg/m^2	30.30 (27.40; 33.30)	29.10 (26.50; 34.83)	0.9
Waist circumference, cm	105.0 (92.0; 110.0)	105.0 (100.3; 118.0)	0.99
Waist-to-hip ratio	0.98 (0.92; 1.11)	0.94 (0.93; 1.06)	0.99
Fat free mass, kg	57.50 (47.00; 61.80)	59.60 (56.70; 62.30)	0.52
Fat mass, adjusted to BMI, kg	36.30 (27.70; 40.00)	30.70 (30.40; 36.70)	0.99
Skeletal muscle mass, kg	24.30 (18.00; 27.80)	26.80 (25.80; 29.20)	0.62
Fasting glycaemia, mmol/L	5.80 (5.15;6.00)	5.70 (5.30;6.33)	0.96
Postprandial glycaemia, mmol/L	6.6 (5.3;7.9)	7.8 (6.6;7.9)	0.09

Fasting insulin, μU/mL	6.5 (4.9;8.6)	5.3 (4.5;7.5557)	0.7
Postprandial insulin, μU/mL	14.1 (11.87;18.1)	21.6 (9.7;33.5)	0.7
HbA1c, %	5.7 (5.5;6.8)	6.8 (6.1;8.5)	0.07
Total cholesterol, mmol/L	3.68 (3.01;4.31)	3.87 (2.88;4.52)	0.22
Triacylglycerols, mmol/L	1.14 (0.90;1.43)	1.35 (1.23;1.85)	0.13
HDL, mmol/L	1.04 (0.95;1.18)	1.04 (0.85;1.30)	0.93
LDL, mmol/L	2.00 (1.55;2.40)	1.92 (1.62;2.55)	0.9
Serum adiponectin, adjusted to BWI, μg/mL	8.07 (4.76;10.60)	6.26 (5.54;6.46)	0.3
Serum leptin adjusted to BWI and to gender, ng/mL	13.96 (11.01;20.77)	24.77 (18.17;38.61)	**0.037**
Adiponectin-to-leptin ratio	0.63 (0.39;0.74)	0.28 (0.17;0.43)	**0.015**

Notes: Gensini score =70.55 points – median value of Gensini score in the sample adjusted to gender
